# Genotype X Environment Interaction and Stability of Quinoa (*Chenopodium quinoa,* Willd.) Genotypes for Yield and Yield-Related Traits in Ethiopia

**DOI:** 10.3390/plants15162436

**Published:** 2026-08-11

**Authors:** Atika Muhhamed, Firew Mekbib, Didier Bazile, Cataldo Pulvento, Berhanu Amsalu Fenta

**Affiliations:** 1 Mechara Agricultural Research Center, Oromia Agricultural Research Institute (OARI), Mechara P.O. Box 81265, Ethiopia; 2 College of Agriculture and Environmental Science, Haramaya University, Dire Dawa P.O. Box 138, Ethiopia; firew.mekbib@gmail.com; 3 CIRAD, UMR SENS, F-34398 Montpellier, France; didier.bazile@cirad.fr; 4 UMR SENS, CIRD, IRD, University Paul Valery Montpellier 3, University Montpellier, F-34090 Montpellier, France; 5 Department of Soil, Food and Plant Sciences, University of Bari (UNIBA), 70126 Bari, Italy; cataldo.pulvento@uniba.it; 6 Melkassa Research Center, Ethiopian Institute of Agricultural Research (EIAR), Adama P.O. Box 436, Ethiopia; 7 College of Agriculture & Environmental Science (CAES), African Sustainable Agriculture Research Institute (ASARI), University Mohammed VI Polytechnic (UM6P), Laayoune 70000, Morocco

**Keywords:** quinoa (*Chenopodium quinoa* Willd.), genotype × environment interaction (GEI), yield and yield-related traits, climate-smart agriculture, agro-ecological adaptation stability analysis, GGE biplot

## Abstract

Agriculture is the pillar of Ethiopia’s economy, employing over 80% of the population. However, it remains highly vulnerable to the impacts of climate change. Promoting climate-smart agricultural practices is therefore critical. Quinoa (*Chenopodium quinoa* Willd.), a highly nutritious pseudo-cereal with broad adaptability and efficient water use, presents a promising alternative crop for Ethiopia’s diverse agro-ecologies. Its tolerance to drought and salinity makes it an ideal candidate for enhancing food and nutritional security under changing climate conditions. Therefore, this study aimed to evaluate the GEI for seed yield and yield-related traits and identify the stable and high-yielding genotypes of quinoa across six agro-ecological locations in Ethiopia—Negelle Arsi, Haramaya, Holeta, Kulumsa, Melkassa, and Ziway—representing three agro-climatic classifications (lowland, midland and highland). Thirteen genotypes were studied using a randomized complete block design (RCBD) with three replications. The G × E interaction main effect revealed significant GEI effects, particularly for plant height, days to maturity, and grain yield. Among the tested genotypes, ‘Brightest Brilliant Rainbow’, ‘Salcedo-INIA-Puno-Peru’, and ‘Multi-Hued’ demonstrated superior performance across multiple environments. Stability analyses were performed using the AMMI, ASV, and GGE biplot approaches. The analyses revealed significant variation among the genotypes for both adaptability and stability. The AMMI results indicated that environmental effects contributed more to yield variability than genotypic or interaction effects. The AMMI model identified four promising genotypes: ‘Salcedo-INIA-Puno-Peru’, ‘Brightest Brilliant Rainbow’, ‘Multi-Hued’, and ‘Bio-bio’. According to the ASV, the most stable genotypes were ‘Amarilla Sacaca’, ‘Amarilla Maranganí’, ‘Bio-bio’, ‘Cherry Vanilla’, ‘Multi-Hued’, and ‘Brightest Brilliant Rainbow’. The GGE biplot identified ‘Brightest Brilliant Rainbow’ and the Holeta location as the most ideal genotype and testing environment, respectively. ‘Multi-Hued’ and ‘Brightest Brilliant Rainbow’ emerged as the winning genotypes in the only defined mega-environment. Notably, ‘Brightest Brilliant Rainbow’ exhibited a high protein content, ranging from 14.8% at Kulumsa to 17.8% at Melkassa. Overall, ‘Brightest Brilliant Rainbow’ and ‘Salcedo-INIA-Puno-Peru’ were identified as high-performing, stable genotypes suitable for cultivation across diverse Ethiopian environments. These findings confirm quinoa’s adaptability to the broader agro-ecologies of Ethiopia and enable selection of stable, widely adapted varieties through the G × E interaction and stability analyses using AMMI, ASV, and a GGE biplot.

## 1. Introduction

Ethiopia covers 1,120,000 square kilometers. The population was estimated at about 112,078,730 in 2019, making it the second most populous country in Africa, after Nigeria [1]. Ethiopia has a wide range of climatic features suitable for different agricultural production systems. As its economy is based on agriculture, which accounts for more than 80% of the total employment [2] and is highly vulnerable to climate change, looking for climate-smart agricultural practices is the best option. In this context, quinoa is recognized as a strategic crop with the potential to contribute to food security and sovereignty due to its high nutritional content, adaptability to different climate and soil conditions, genetic variability and low production cost [3].

Quinoa can grow in 40% to 88% relative humidity and, depending on the variety, at temperatures from 15 to 20 °C and can resist temperatures from −4 °C to 38 °C [4]. The rainfall conditions needed are highly variable between the different cultivars, ranging from 300 to 1000 mm during the growing season and precipitation levels of 50 to 100 mm during the rest of the year [5]. Quinoa can grow in highly acidic and alkaline soils and tolerates soils with different textures and pH [6]. Quinoas’ grain leaves and inflorescences are all sources of high-quality essential amino acids (EAAs), especially lysine, at levels more than those found in proteins obtained from other cereals. That is why it is considered the only plant food that provides EAAs [7]. It also contains saponin, which is used as an industrial raw material [8]. A study that analyzed quinoa’s Injera-making quality showed that the fat, protein and total calorie content of quinoa grain were significantly higher than those of tef grain, but not significantly different in its fiber and ash contents [9]. There are several products derived from quinoa, such as puffed quinoa, flour, pasta, flakes, granola, energy bars, etc. [10]. Quinoa also has medicinal properties that are used for healing in the communities of the Altiplano and valleys. It has applications in beverages, shampoo and soaps [3]. As of 2014, the world’s leading quinoa-producing countries were Peru, Bolivia and Ecuador, producing 41, 38.3 and 0.8 thousand metric tons, respectively [3].

Quinoa (*Chenopodium quinoa* Willd.) is a species of the goose foot genus, a grain crop grown primarily for its edible grain [11]. As a member of the Amaranthaceae family, it is related to and resembles amaranth [3]. Quinoa is a pseudo-cereal annual plant. It originated in the Andean regions of Bolivia, Peru, Ecuador, Chile and Colombia, where it was domesticated 3000 to 4000 years ago for human consumption [8]. The genus Chenopodium is the largest in the family Chenopodiaceae and has a worldwide distribution, with about 250 species. It is a species with a broad center of origin and multiple diversifications. The five groups of quinoas according to [12,13] are: sea level quinoas, valley quinoas, Altiplano quinoas, salt flat quinoas and quinoas of the Yungas. There are 59 gene banks in 30 countries which have conserved 16,422 accessions and their wild relatives (*C*. *quinoa*, *C*. *album*, *C*. *berlandieri*, *C*. *hircinum*, *C*. *petiolare*, *C*. *murale* and *Chenopodium* sp.). The Andean region gene bank holds 88% of the crop’s conserved accessions [14,15]. Agromorphological and molecular characterizations of the collection have been made. The growth habit, plant color, grain color and shape, panicle shape and density, grain yield per plant, crop cycle and grain diameter were used for the agromorphological characterization, while seventeen microsatellite primers and ISSR markers were used for the typing [14]. Quinoa is an Allotetraploid (2n = 4x = 36) with a base chromosome number of x = 9. Genetic sequencing revealed that its genome is derived from two distinct diploid ancestors (carrying approximately 18 chromosomes each), which contributes to its broad genetic diversity and high physiological plasticity [16]. There are a wide range of breeding objectives for quinoa, such as breeding for a short cycle, earliness, low saponin content, abiotic and biotic stresses and yield [16]. DNA markers and linkage maps are important tools for crop improvement and germplasm conservation. RASP and ISSR markers have been used in the past for marking genetic variations in quinoa [17]. Microsatellite markers have also been also used to identify the genetic diversity between quinoa and other *Chenopodium* species [18].

Recent studies have identified the genomic regions associated with grain yield and yield-related traits, providing useful tools for speeding up the development of high-yielding varieties. Even though considerable progress has been achieved in quinoa genomics and molecular breeding, phenotypic evaluation across diverse environments remains essential because grain yield is strongly influenced by the genotype × environment interaction. In Ethiopia, limited information exists regarding the adaptability and stability of quinoa genotypes under different agro-ecological conditions. Thus, conducting a G × E interaction analysis helps the researcher to select the superior genotype in terms of yield and stability. This experiment was conducted with the objectives of estimating the effect of genotype, environment and GEI on the seed yield and yield-related traits and to determine the stability of quinoa genotypes for grain yield and yield-related traits across environments.

## 2. Results

The results obtained from the data collected from thirteen genotypes at six locations were subjected to an analysis of variance for each location and across locations, to understand performance of genotypes for their grain yield across environments, particularly the genotypes’ mean performance for yield-related traits and yield. Furthermore, an AMMI analysis, GGE biplot analysis for grain yield, ASV and nutritional content analysis were applied. As a result, the analysis determined that the environmental effect is higher than that of both the genotypes and their interactions. In addition, high-yielder and stable genotypes were identified. Also, nutritional content of the genotypes was determined. Therefore, this paper discusses in detail the findings listed above.

### 2.1. Performance of Thirteen Varieties in Six Environments

#### 2.1.1. Mean Performance of Thirteen Genotypes for Yield and Yield-Related Traits

The results of a combined mean for yield and yield-related traits showed that there were highly significant (*p* < 0.01) differences in plant height, days to maturity and grain yield, while there was a significance difference (*p* < 0.05) in the rest of tested traits (Table 1).

Kancolla (104.47 cm) had the highest plant height, whereas Amarilla Sacaca (87.22 cm) had the lowest plant height. Bio-bio (17.78), Salcedo INIA (17.77), Amarilla Maranganí (17.71), Blanca de Junín (17.69), and QQ74 (17.68) recorded the highest number of primary branches per plant, while Cherry Vanilla (14.70) had the lowest number. The longest panicle length was produced by Amaranths (Golden) (SC) (24.91 cm), whereas Amarilla Sacaca (19.39 cm) had the shortest panicle length.

In terms of panicle width, Blanca de Junín (13.11 cm), Amarilla Maranganí (14.66 cm), Salcedo INIA (13.05 cm), Kancolla (13.81 cm), QQ74 (13.79 cm), Titicaca (14.49 cm), and Amaranths (Golden) (SC) (12.08 cm) had wider panicles; Cherry Vanilla (7.72 cm) showed the narrowest panicle width. Amaranths (Golden) (SC) (106.84 days) was late-maturing compared with Amarilla Sacaca (92.88 days), Brightest Brilliant Rainbow (90.65 days), and Salcedo-INTA-Puno-Peru (92.21 days), which were early-maturing.

Blanca de Junín (28.31) had a higher number of panicles per plant than QQ74 (22.55). Brightest Brilliant Rainbow (44.34) recorded the least number of stand counts at harvest, while Amaranths (Golden) (SC) (54.86) had the largest stand count. The mean separation indicated that Brightest Brilliant Rainbow (1967.64), Salcedo-INTA-Puno-Peru (1777.53) and Multi-Hued (1671.24) were the high yielders compared to all of the remaining tested genotypes, while Titicaca (1113.48) and Amaranths (Golden) (SC) (1225.96) were the lowest yielders of all. The genotypes’ average grain yield across environments ranged from Titicaca at 1113.48 kg and Amaranths (Golden) (SC) at 1225.96 kg to Brightest Brilliant Rainbow at 1967.64 kg, Salcedo-INTA-Puno-Peru at 1777.53 kg and Multi-Hued at 1671.24 kg (Table 1).

The average grain yield was found to be 630.9 kg to 2857.7 kg per hectare. The growth habit of Blanca de Junín, Multi-Hued and Amaranths (Golden) was branched to the bottom third, but the other genotypes were branched to the second third. Blanca de Junín, Salcedo INIA, Brightest Brilliant Rainbow, Multi-Hued and Amaranths (Golden) had a compact panicle density and the other eight genotypes had intermediate panicle density. There was no Lax. Cherry Vanilla had an Amarantiform panicle Shape, Multi-Hued had Glomerulate and Amaranths (Golden) had Amarantiform panicle shape. The rest of ten genotypes had an intermediate panicle shape (Appendix A.1).

#### 2.1.2. Performance of Genotypes for Their Grain Yield Across Environments

The different quinoa genotypes exhibited distinct grain yield performances across the test environments. At Melkassa, Multi-Hued, Brightest Brilliant Rainbow, Blanca de Junín, and Bio-bio performed well; at Ziway, Multi-Hued, Kancolla, and Bio-bio were the top performers. At Negelle, Arsi, Salcedo-INTA-Puno-Peru, Brightest Brilliant Rainbow, and Multi-Hued showed good performance while, at Kulumsa, the high-performing genotypes were Brightest Brilliant Rainbow, Salcedo-INTA-Puno-Peru, and Salcedo INIA. In the fifth environment, Holeta, the best performers were Salcedo-INTA-Puno-Peru, Brightest Brilliant Rainbow, and Salcedo INIA. Finally, at Haramaya, Brightest Brilliant Rainbow, Salcedo-INTA-Puno-Peru, and Multi-Hued were the top genotypes (Table 2). Overall, the results indicate that Brightest Brilliant Rainbow performed well in five environments, except at Ziway, whereas Salcedo-INTA-Puno-Peru and Multi-Hued performed well in four environments each.

The grain yield ranged from 911 kgha^−1^ at Melkassa to 1970 kg ha^−1^ at Kulumsa (Table 3). Kulumsa was the best environment for the quinoa genotypes, showing the highest environmental mean of 1970 kg ha^−1^. Overall, Brightest Brilliant Rainbow, Salcedo-INTA-Puno-Peru, and Multi-Hued ranked among the top three grain yields in most environments.

#### 2.1.3. Analysis of Variance for Each Environment

The results suggest considerable variability among the tested genotypes in response to environmental conditions. Most of the tested yield and yield-related components at each location show either a highly significant difference (*p* < 0.01%) or significant difference (*p* < 0.05%) among the quinoa genotypes (Table 3). The grain yield shows highly significant differences at Ziway, Negelle Arsi, Kulumsa, and Holata. In contrast, the differences at Melkassa and Haramaya are not significant (Table 3). For the yield-related traits, the plant height shows a highly significant difference at Kulumsa and a significant difference at Haramaya. The panicle length and panicle width show a significant difference at Ziway; number of panicles per plant is significant at both Negelle Arsi and Haramaya. The stand count at harvest is significant at Ziway, whereas the days to maturity is significant at Melkassa and highly significant at Kulumsa (Table 3).

#### 2.1.4. Combined Analysis of Variance Across Locations

At *p* < 0.01, for the main effect of environment, the plant height, number of primary branches per plant, panicle length, panicle width, number of panicles per plant, stand count at harvest, days to maturity, and grain yield all showed highly significant differences among the treatments (Table 4).

Meanwhile, for the main effect of genotype, the number of primary branches per plant showed significance; however, the days to maturity and grain yield showed highly significant differences at *p* < 0.01%, whereas number of primary branches per plant was significant for both the environment and genotype as main effects. For their interaction, the main effect of G × E, the plant height and days to maturity showed a significant difference and the grain yield showed a highly significant difference (Table 4).

### 2.2. Additive Main Effects and Multiplicative Interaction (AMMI) Analysis

The grain yield analysis done using the AMMI model showed the pattern and relationship of genotype, environment and their interactions. As seen from the total sum of squares in Table 5, the genotype accounts for 16.46, the environment for 66.21, and their interactions account for 8.87. The largest portion of total sum of squares belongs to the environment, showing that most of the grain yield variation is caused by the environment.

After, the data for each trait were subjected to a combined analysis of variance to estimate the effects of environment, genotype and genotype x environment interaction. An AMMI analysis was employed to conduct the analysis of additive main and multiplicative effects for the genotype × environment interaction. As a result, the IPCA1 was highly significant although the IPCA2 was non-significant. The IPCA1 captured 64.56 percent of the interaction, which was bigger than the IPCA2 at 17.99, and all together added up to 82.54 percent (Table 5).

#### 2.2.1. First Four AMMI-Selected Genotypes

The genotype Salcedo-INTA-Puno-Peru (G12) was selected in all six environments (Table 6), being the second-best genotype at Negelle Arsi, Haramaya, Kulumsa and Holeta, while being the third- and fourth-best at Ziway and Melkassa, respectively. The genotype Brightest Brilliant Rainbow (G6) was selected in five environments (Melkassa, Negelle Arsi, Haramaya, Kulumsa and Holeta), ranking second at Melkassa and first in the rest of four environments listed above. Multi-Hued (G9) become the third AMMI-selected genotype by being selected in four environments. At Melkassa and Ziway, it was selected as the first genotype, while at Negelle Arsi and Haramaya it was selected as the third-best genotype. Finally, Bio-bio (G7) was selected as the fourth-best AMMI-selected genotype and performed well in three environments, being second at Ziway, third at Melkassa and fourth at Negelle Arsi.

#### 2.2.2. AMMI Biplot for Grain Yield

**Figure 1:**
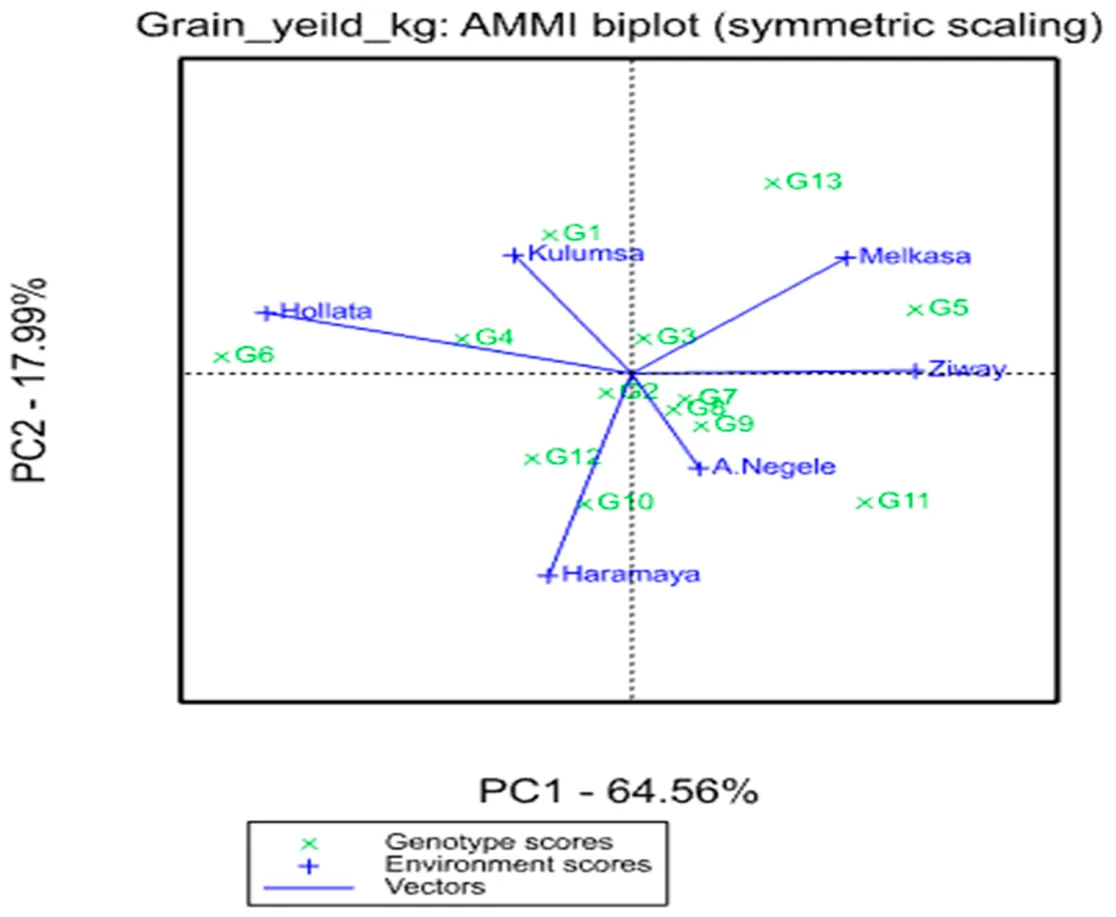
AMMI biplot of grain yield for thirteen genotypes evaluated in six locations. Note: G1 = Blanca de Junín; G2 = Amarilla Sacaca; G3 = Amarillla Maranganí; G4 = Salcedo INIA; G5 = Kancolla; G6 = Brightest Brilliant Rainbow; G7 = Bio-bio; G8 = Cherry Vanilla; G9 = Multi-Hued; G10 = QQ74; G11 = Titicaca; G12 = Salcedo-INTA-Puno-Peru; G13 = Amaranthus.

According to the AMMI biplot for grain yield, Haramaya, Melkassa, Ziway and Holeta are distant from the origin. While Kulumsa and Negelle Arsi are near to the origin relatively, they contribute less to the instability of the genotypes (Figure 1). Genotypes G3 (Amarillla Maranganí), G7 (Bio-bio), G2 (Amarilla Sacaca), G9 (Multi-Hued), G8 (Cherry Vanilla), and G12 (Salcedo-INTA-Puno-Peru) are near to the origin that shows minimal interaction with the environment. These genotypes’ minimal interaction implies that they are stable too. In contrary, the genotypes G6 (Brightest Brilliant Rainbow), G1 (Blanca de Junín), G13 (Amaranths (Golden)), G5 (Kancolla), G11 (Titicaca), G4 (Salcedo INIA) and G10 (QQ74) are far from the origin. Therefore, they are less stable, and they have a high interaction with the test environment.

### 2.3. GGE Biplot Analysis of Grain Yield of Thirteen Genotypes Evaluated Across Six Locations

#### 2.3.1. Mean Performance and Stability of the Genotype

**Figure 2:**
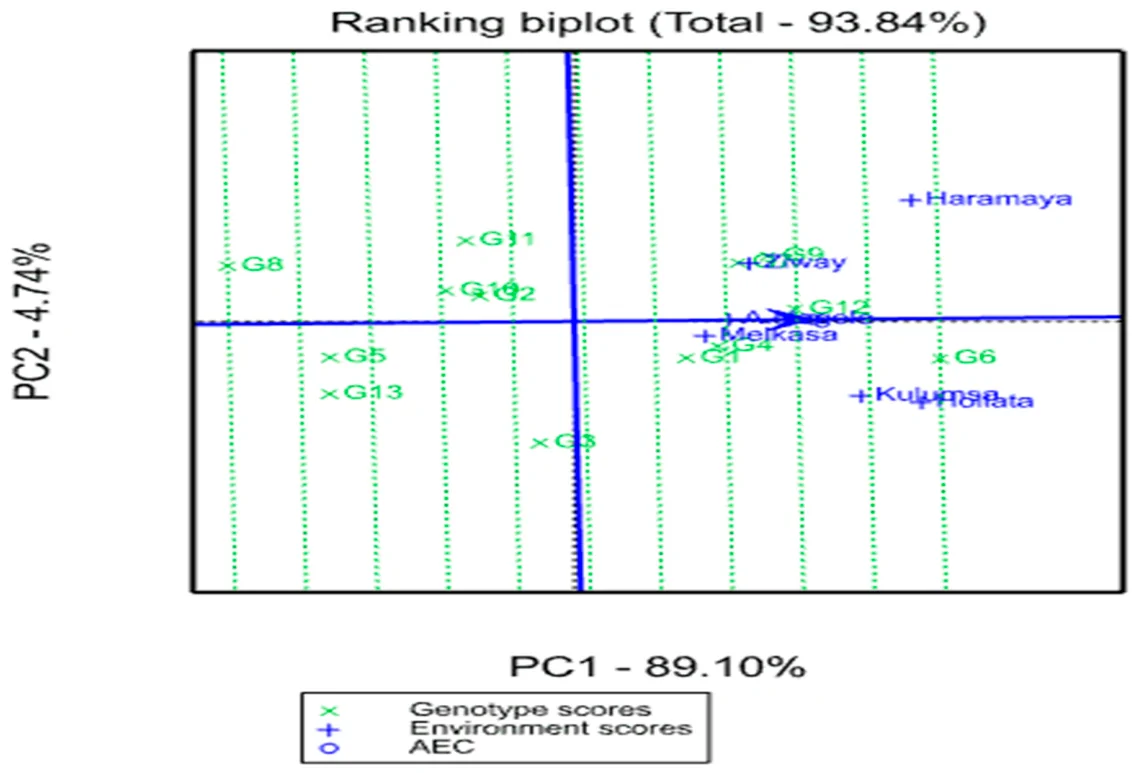
GGE biplot showing the mean performance and stability of thirteen genotypes evaluated across six locations.

Accordingly, the high-yielding genotypes are G12 (Salcedo-INTA-Puno-Peru) and G9 (Multi-Hued), since the dotted line that both are found on lies exactly on the arrow passing along the *X*-axis (AEC), and the lowest genotype is G8 (Cherry Vanilla) (Figure 2).

The line that passes through the biplot origin perpendicular to the *X*-axis separates the genotypes that yield above the means. In this study, it separates the G12 (Salcedo-INTA-Puno-Peru), G9 (Multi-Hued), G6 (Brightest Brilliant Rainbow), G7 (Bio-bio), G4 (Salcedo INIA) and G1 (Blanca de Junín) genotypes, which yield above the mean, and G3 (Amarillla Maranganí), G2 (Amarilla Sacaca), G11 (Titicaca), G10 (QQ74), G5 (Kancolla), G13 (Amaranths (Golden)), and G8 (Cherry Vanilla), which yield below the mean.

The most stable genotypes are those showing the shortest distance from the Average Environment Coordination (AEC). Hence, genotypes G5 (Kancolla), G2 (Amarilla Sacaca), G10 (QQ74), G12 (Salcedo-INTA-Puno-Peru), G4 (Salcedo INIA), G1 (Blanca de Junín) and G6 (Brightest Brilliant Rainbow), being near to the AEC, they are the stable ones. Genotypes G8 (Cherry Vanilla), G11 (Titicaca), G13 (Amaranths (Golden)) and G3 (Amarillla Maranganí) are the unstable genotypes across the environments, and they contribute to the genotype-by-environment interaction since they have the longest distance from the Average Environment Coordination (AEC). Finally, considering both the mean yield and stability together, genotypes G12 (Salcedo-INTA-Puno-Peru), G4 (Salcedo INIA), G1 (Blanca de Junín) and G6 (Brightest Brilliant Rainbow) fulfill both criteria for being the best genotypes.

#### 2.3.2. Ranking Genotypes Relative to the Ideal Genotype

**Figure 3:**
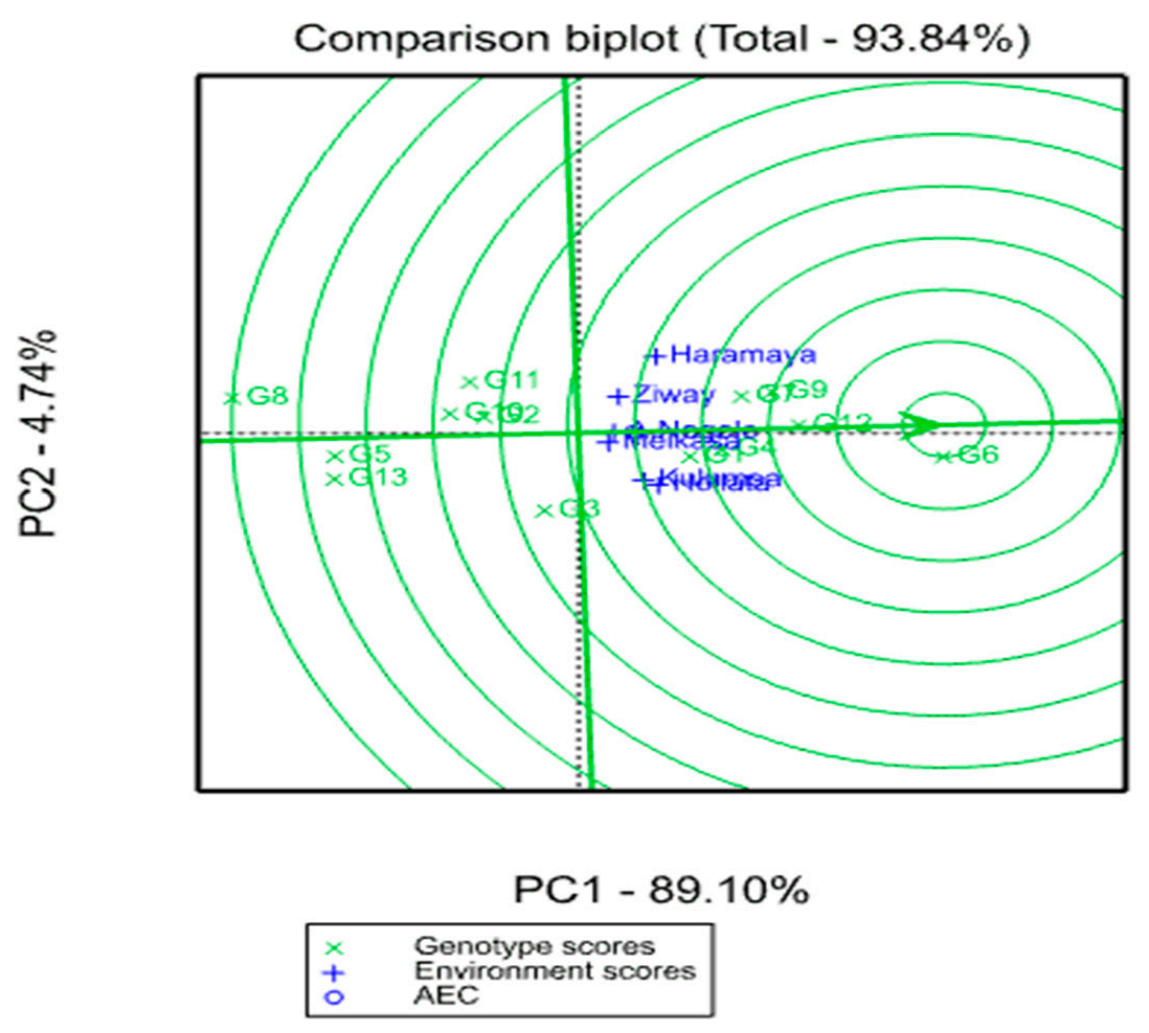
GGE biplot showing the ranking of thirteen genotypes relative to the ideal genotype across six locations.

Brightest Brilliant Rainbow (G6), followed by Salcedo-INTA-Puno-Peru (G12) or a desirable genotype (Figure 3), are the ideal genotypes. Desirable genotypes are those located closer to the ideal genotypes. The farther a genotype is, the less ideal it is (Figure 3). Accordingly, the ranking of desirable genotypes is G9 (Multi-Hued), G7 (Bio-bio), G4 (Salcedo INIA) and G1 (Blanca de Junín); G8 (Cherry Vanilla), G5 (Kancolla) and G13 (Amaranths (Golden)) are very far from the ideal genotype and are low yielders and have unstable genotypes.

#### 2.3.3. Ranking Environments Relative to the Ideal Environment

**Figure 4:**
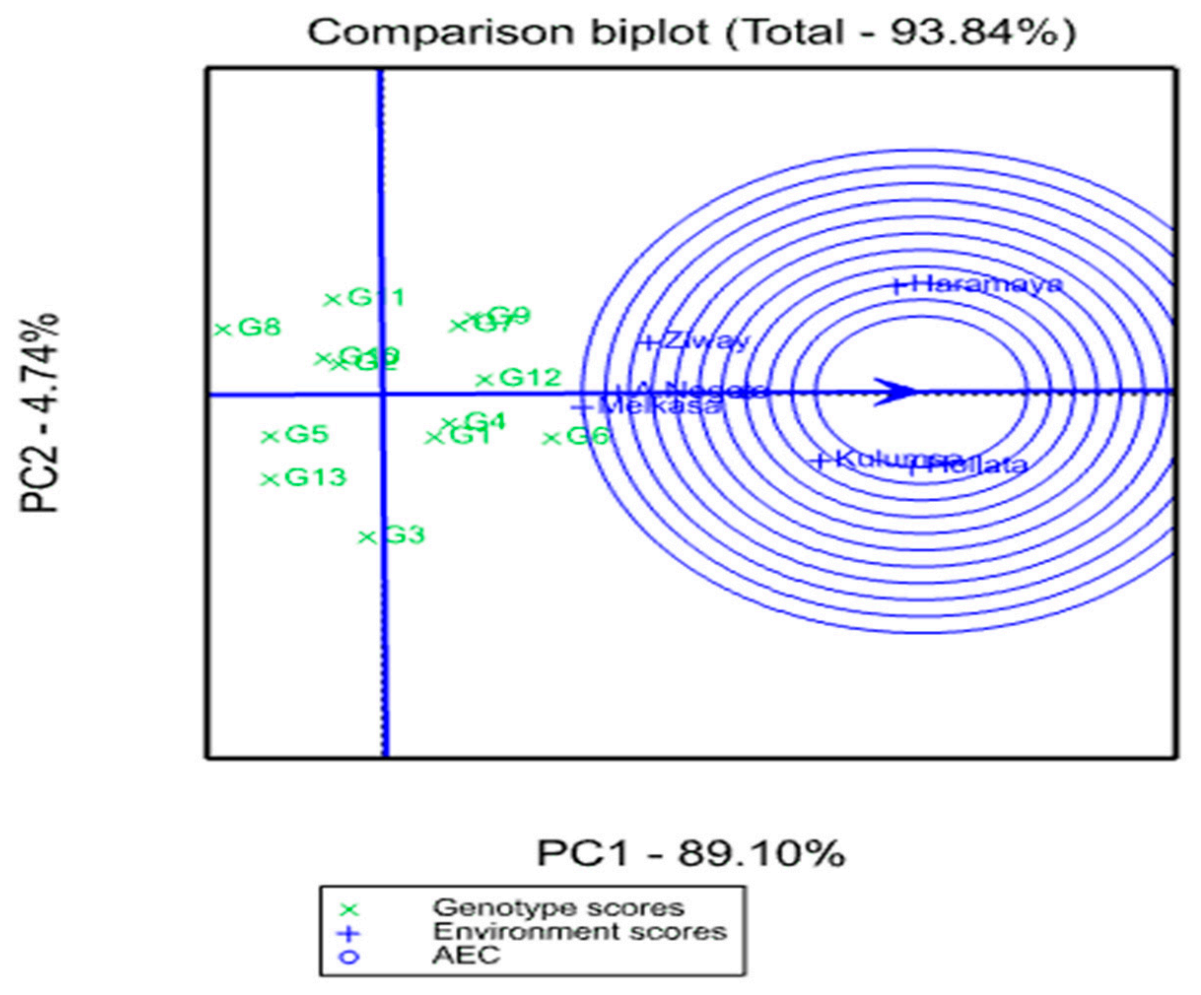
GGE biplot for ranking environments relative to the ideal environment for thirteen genotypes evaluated across six locations.

Holeta is the ideal environment, followed by Kulumsa and Haramaya, which are found at the same distance from the ideal environment, and both can be said to be desirable environments. Melkassa is the farthest environment and is not representative. Therefore Holeta, Kulumsa and Haramaya are regarded as the most suitable for selecting widely adapted genotypes (Figure 4).

#### 2.3.4. Which-Won-Where Pattern of GGE Biplot

**Figure 5:**
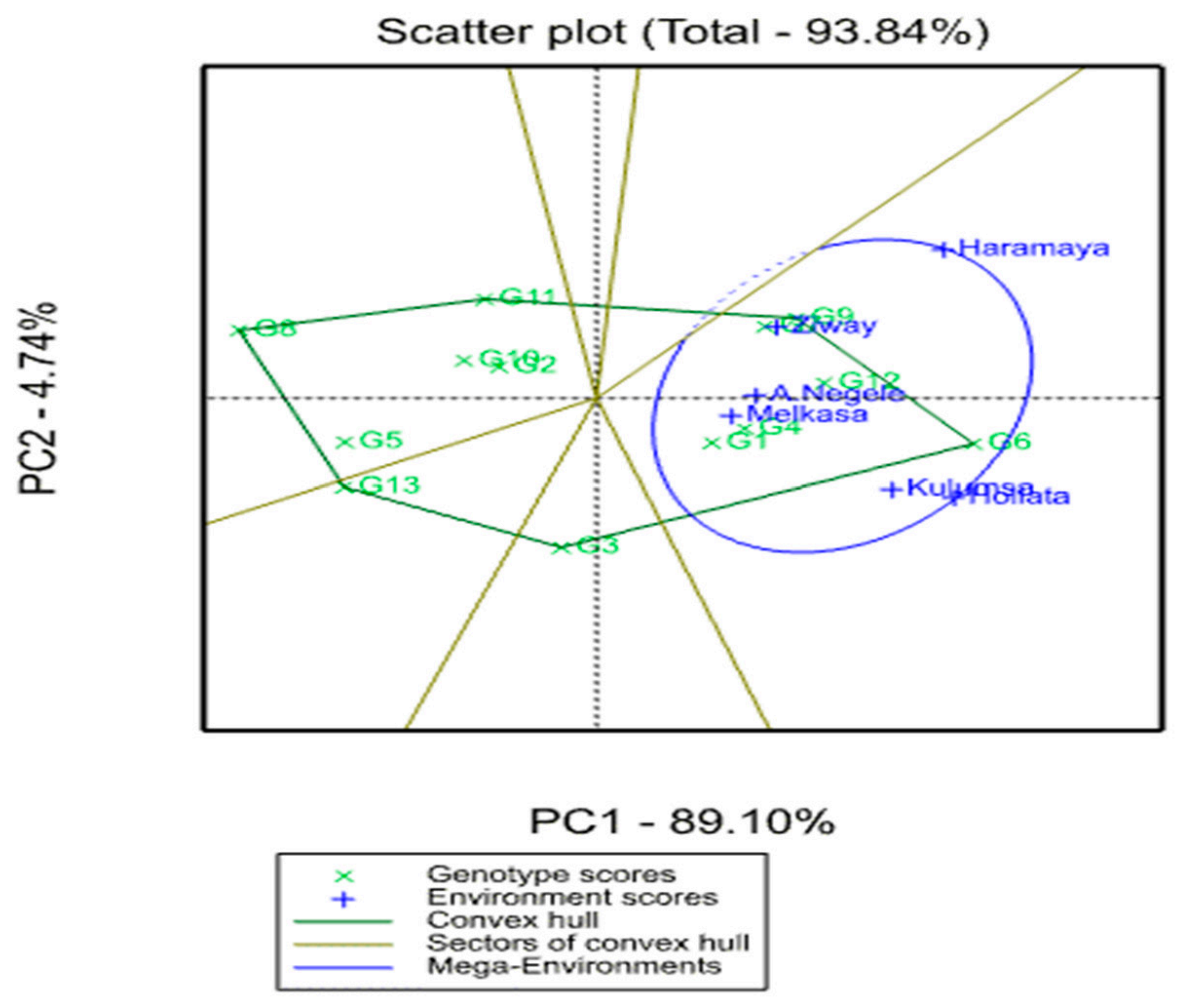
GGE biplot for mega-environment analysis of thirteen genotypes tested across six experimental sites.

G9 (Multi-Hued), G6 (Brightest Brilliant Rainbow), G11 (Titicaca), G8 (Cherry Vanilla), G13 (Amaranths (Golden)) and G3 (Amarillla Maranganí) are vertex genotypes (Figure 1); with this result, there is only one mega-environment containing all six environments that contains the two winning genotypes, G9 (Multi-Hued) and G6 (Brightest Brilliant Rainbow), found on the vertex. These two vertex genotypes are the winning genotypes, with better yield performances than any other genotypes included in the respective sector. The environments within the same sector share the same winning genotype, so as there is only one mega-environment, all environments share the same winning genotypes, but the environments within the mega-environment also have their own winning genotypes in their sector, which is the genotype closest to them. Therefore, G1 (Blanca de Junín) and G4 (Salcedo INIA) are too near to Melkassa, so these genotypes are the winners at Melkassa rather than another environment, and G12 (Salcedo-INTA-Puno-Peru) and G9 (Multi-Hued) are the winners at Negelle Arsi and Ziway (Figure 5).

The vertex genotypes G8 (Cherry Vanilla), G13 (Amaranths (Golden)) and G3 (Amarillla Maranganí) are not the best in any of the test environments since there is no environment that falls into their quadrant and/or their sectors.

### 2.4. AMMI Stability Value (ASV)

Regarding the ASV results, Amarilla Sacaca (4.44), Amarillla Maranganí (26.63), Bio-bio (15.53), Cherry Vanilla (13.24), Multi-Hued (32.19), and Brightest Brilliant Rainbow (32.79) have the lower ASVs, so they can be called the most stable genotypes (Table 7).

As the most stable genotype would not inevitably be a high yielder, the stability would not be the only criteria for selection. Therefore, there is a need for an approach that incorporates both the mean grain yield and stability in a single standard. The YSI, which incorporates both the mean grain yield and stability at once, is used to figure out the yield and stability of the genotype. The lower the YSI is, the more stable and higher yielder the genotype is. As a result, Brightest Brilliant Rainbow (7), Bio-bio (7), Multi-Hued (8) and Salcedo-INTA-Puno-Peru (8) are considered stable and high-yielder genotypes. Cherry Vanilla (13), Amaranths (Golden) (23), Kancolla (23), and Titicaca are unstable and produce less yield than the mean yield (Table 7).

### 2.5. Nutritional Content of Quinoa

To examine the nutritional quality, four quinoa genotypes were selected as representative varieties and evaluated for their nutritional contents. These genotypes were Brightest Brilliant Rainbow (BBR), Titicaca, Amaranth, and Kancolla, grown at five locations: Melkassa, Holeta, Ziway, Kulumsa, and Negelle Arsi.

Based on the results of this study, the protein content ranged from 17.8 to 14.8. The highest protein content (17.8) was recorded for Brightest Brilliant Rainbow grown at Melkassa, followed by Titicaca (17.3) grown at Holeta, and Amaranth (17.0) grown at Melkassa and Negelle Arsi. However, Brightest Brilliant Rainbow grown at Kulumsa showed the lowest protein content (14.8). The essential amino acids were also assessed (Appendix A.2), as they are crucial for growth and development. This study therefore focuses on the essential amino acids. As a result, Brightest Brilliant Rainbow grown at Ziway recorded the highest threonine content (0.88) among the tested genotypes, with values ranging from 0.88 to 0.56. The valine content ranged from 1.05 in Brightest Brilliant Rainbow grown at Ziway to 0.79 in Titicaca grown at Holeta. The amaranth grown at Negelle Arsi contained the highest methionine content (0.29), whereas Brightest Brilliant Rainbow grown at Kulumsa had the lowest methionine content (0.14). The amaranth grown at Negelle Arsi also showed the highest phenylalanine (0.73) and lysine (1.34) contents. In contrast, Brightest Brilliant Rainbow grown at Holata and Titicaca grown at Melkassa recorded the lowest phenylalanine (0.54) and lysine (0.79) contents, respectively. For isoleucine, Kancolla grown at Holeta recorded 1.04, while Brightest Brilliant Rainbow grown at Negelle Arsi had the lowest isoleucine content (0.68). The highest leucine content (1.29) was found in Titicaca grown at Kulumsa, whereas the lowest (0.98) was recorded in amaranth grown at Holeta (Appendix A.2).

## 3. Discussion

### 3.1. Performance of Thirteen Quinoa Genotypes Across Six Environments for Yield and Yield-Related Traits

The observed significant differences in plant height, days to maturity, and grain yield confirm the presence of genetic variability among the tested quinoa genotypes [19]. The plant height of the cultivars ranged from 34.85 to 127.35 cm, with Amarilla de Marangani being the tallest (up to 2.30 m). In contrast, Puno and Titicaca exhibited intermediate heights. According to [20], plant height generally ranges from 66.8 to 116.4 cm. In this study, Rainbow showed heights of 106.0 cm and 112.5 cm, while Cherry Vanilla recorded 78.5 cm and 111 cm at Erzurum and Igdir, respectively. Likewise, ref. [21] reported that plant height ranged from 49.4 to 167.9 cm across two years of data. The tall stature of Kancolla and the comparatively low height of Amarilla Sacaca fall within the ranges reported by earlier studies, suggesting that both the genotype and environment contribute to plant architecture.

The panicle length varied from 9.7 cm to 22.5 cm [21]. The days to maturity ranged from 107 to 158 days for the quinoa varieties grown at two locations (Erzurum and Igdir) [20]. The Rainbow genotype matured in 121 and 142 days, whereas Cherry Vanilla matured in 122 and 142 days at Erzurum and Igdir, respectively [20]. In addition, the days to maturity varied from 72 to 123 days in this study, while in the Andean region it has been reported as 110 to 190 days [21]. Early-maturing genotypes, such as Brightest Brilliant Rainbow and Salcedo-INTA-Puno-Peru, are valuable for areas with short rainy seasons, whereas late-maturing types, such as Amaranths (Golden) (SC), may be more suitable for regions with extended growing periods.

The grain yield is the trait that shows a high genotype-by-environment interaction (GEI) [22]. Studies conducted in different locations worldwide have reported average yields ranging from 250 to 5000 kg per hectare [20]. For example, ref. [20] reported that the Rainbow cultivar yielded 931.6 kg per hectare, while Titicaca ranked first with a grain yield of 25.19 g per plant [23]. The top-performing genotypes that exhibited favorable traits—such as compact panicle density and branching patterns conducive to higher yield—were Brightest Brilliant Rainbow, Salcedo-INTA-Puno-Peru, and Multi-Hued. Overall, these results support the selection of the high-performing and early-maturing genotypes for breeding programs aimed at improving quinoa adaptation and yield across diverse Ethiopian agro-ecologies.

A study by [21] showed that quinoa yield is strongly influenced by environmental factors and the G × E interaction. This interaction reflects the differential adaptability and stability of genotypes under varied environmental conditions. Genotypes with compact panicle density and a branched-to-the-bottom-third growth habit, such as Brightest Brilliant Rainbow and Multi-Hued, adapted well across different environments.

Regarding the environmental effects, Kulumsa was the highest-yielding environment among the six locations. This may be due to the adequate and timely rainfall it received. This finding is consistent with [24], who reported that acceptable yields can be obtained with rainfall of 100–200 mm. However, growth is optimal when rainfall is well distributed during early growth and development, followed by drier conditions during seed maturation and harvest [24].

The highly significant differences observed in the combined analysis highlight the influence of environmental variability on genotype performance. This significant G × E interaction—particularly for grain yield—indicates that the genotypes responded differently across environments. The highly significant G × E interaction suggests that genotypes may be selected for adaptation to specific environments. As reported by [25,26], a strong G × E interaction was observed for grain yield and primarily for the yield components (biomass and harvest index), driven by differences among the cultivars and environmental variability. Similarly, in [27]’s report, the combined analysis of variance revealed significant effects of environment, genotype, and their interaction on fodder yield of dual-purpose barley genotypes. Another study also reported significant differences for genotype and the genotype × year interaction [21]. A combined analysis of grain yield for six rice genotypes showed highly significant effects (*p* < 0.001) for genotype, environment, and their interaction. Overall, these findings confirm that such interactions must be considered carefully in genotype selection; otherwise, they may pose a challenge in crop improvement programs.

In this experiment, the difference in grain yields across the different locations indicates a G X E interaction, which is consistent with the findings of previous studies, such as [28,29,30], which reported substantial variations in the grain yield performance of common bean genotypes across environments. A strong G × E interaction was also observed for the grain yield and yield components (biomass and harvest index), reflecting the combined effects of genetic differences among genotypes and environmental variation across locations [26].

### 3.2. Additive Main Effects and Multiplicative Interaction (AMMI) and AMMI Biplot Analysis for Grain Yield

An ANOVA can quantify interactions and describe the main effects; however, it is insufficient for fully explaining the genotype × environment interaction (GEI). Therefore, the AMMI model (Additive Main Effects and Multiplicative Interaction) is useful for understanding the GEI because it helps summarize the patterns and relationships among the genotype, environment, and their interaction [31,32]. The findings of [33,34] showed that 90.76% of the total sum of squares was attributed to environmental effects, 2.5% to genotypic effects, and 7.12% to genotype × environment interaction effects. These results indicate that environmental variation contributed most to the differences observed in grain yield. In contrast, ref. [35] reported that rice grain yield variation was mainly affected by the genotype (76.51%), followed by the environment (12.49%) and GEI (10.21%), indicating a greater genotypic effect. Similarly, ref. [27] reported that the effects of location (47.3%), genotype (6.5%), and genotype × environment interaction (37.8%) were highly significant. They concluded that significant G × E interactions indicated the presence of both crossover and non-crossover interactions. Consequently, an analysis of variance followed by a principal component analysis (PCA) of the residuals was recommended for evaluating the fodder yield in dual-purpose barley.

The AMMI model partitions the interaction sum of squares into interaction principal component (IPC) axes and residuals [31,32]. Several studies conducted on different crops have partitioned the G × E interaction into IPCAs. The results of the present study show that the environmental effect was greater than the genotype and interaction effects, indicating substantial environmental diversity. Furthermore, the G × E interaction was partitioned into IPCA1 and IPCA2, with IPCA1 accounting for 93% of the total G × E interaction sum of squares among 15 finger millet genotypes [36]. Likewise, ref. [37] found that the AMMI PCA1 and PCA2 were significant, explaining 35% and 29.5% of the G × E variation in hybrid and inbred maize trials, respectively. In another AMMI analysis, ref. [33] reported that the cumulative mean sum of squares for PCA1 and PCA2 accounted for 78.64% of the total GEI, which was greater than the genotype sum of squares, and both the PCA1 and PCA2 were significant at *p* = 0.01. Therefore, they concluded that the AMMI model with only two interaction principal component axes was the best predictive model [33]. An AMMI biplot also reveals the stability or instability of a genotype [37]. One study used an AMMI biplot for grain yield to evaluate 17 tropical maize inbreds in 12 stress and non-stress environments [36]. It also used an AMMI biplot for evaluating 15 finger millet genotypes in three environments.

### 3.3. GGE Biplot Analysis for Grain Yield

The mean yield performance of genotypes across environments can be approximated or estimated using the Average Environment Coordination (AEC) [38]. Similarly, ref. [39] used the AEC to identify the mean yield performance of genotypes across environments in sorghum multi-location trials. Genotypes with higher PCA1 scores were identified as high yielders, whereas genotypes with lower PCA1 scores were considered low yielders [33]. The ideal genotype is the one with the highest yield of all, and it is absolutely stable, located in the biplot’s first concentric circle and indicated by the arrow found in the circle [40]. Ref. [41], a study on bread wheat, and [42], a study on basmati rice, with two years of data, used the concentric circle to get the ideal genotype. The ideal test environment is the one that is able to discriminate between genotypes based on the genotypic main effect and can represent the overall environment, so that it is called the representative environment [43]. It is identified by the small circle located in the center of concentric circles and an arrow. Ref. [41] identified the desirable environment for bread wheat and [42] used a GGE biplot to identify the representative environment for basmati rice.

A GGE biplot is also an effective tool for a mega-environment analysis (e.g., “which-won-where” pattern), to recommend specific genotypes for specific mega-environments [38]. The genotypes that are near the origin of the plot are more stable than the vertex genotypes [33]. They are the best in the environment, lying within their respective sector in a polygon view of the GGE biplot [43]; thus, these genotypes are considered specifically adapted. The genotypes close to the origin of axes have a wider adaptation [44]. According to [43], genotypes within the polygon and nearer to the origin of the axes have a wider adaptation and lesser response to environmental variation.

### 3.4. AMMI Stability Value (ASV)

AMMI stability value (ASV) statistics were developed by [45] to quantify and rank genotypes on the basis of their yield stability. An AMMI2 model with IPCA1 and IPCA2 scores and an ASV with its ranking was used on South Africa maize hybrids to discriminate between 23 hybrids [46].

The AMMI and GGE biplot analyses revealed that environmental effects contributed the largest proportion of the total variation, highlighting the importance of environmental conditions in determining genotype performance. Brightest Brilliant Rainbow exhibited greater deviation from stability in the AMMI biplot while, in the ASV and YSI analyses, it was a stable and high-yielding genotype. This divergence arises because the AMMI biplot and ASV focus strictly on environmental instability, whereas the YSI incorporates both yield and stability to provide a more comprehensive assessment. Multi-Hued, Amarilla Sacaca, Amarillla Maranganí, Bio-bio, Cherry Vanilla, and Brightest Brilliant Rainbow possessed desirable stability characteristics across environments. Overall, Brightest Brilliant Rainbow and Salcedo-INTA-Puno-Peru were identified as the most stable and widely adapted genotypes across environments, whereas Holeta and Kulumsa were recognized as favorable and representative testing environments. These findings emphasize the importance of GEI and stability analyses in identifying adaptable and high-performing quinoa genotypes for diverse production environments.

### 3.5. Nutritional Content of Quinoa

The quinoa grain is a rich source of high-quality essential amino acids (EAAs), particularly lysine, at levels higher than those found in proteins from most other cereals [47]. According to [48], the balance of essential amino acids in quinoa protein compares favorably with that of milk protein. For this reason, quinoa is considered one of the few plant foods capable of providing a well-balanced profile of essential amino acids [7]. Depending on the variety, quinoa protein content ranges from 13.81% to 21.9%. Studies conducted in Morocco and Egypt have showed that the quinoa protein content in Africa ranges from 12.5% to 14.1% [47]. Similarly, refs. [49] reported quinoa protein contents ranging from 13.8% to 16.5% and from 13.9% to 17%, respectively.

Furthermore, ref. [50] evaluated the protein content of three quinoa varieties grown in Kenya, Uganda, and Zambia, and found that their protein contents were within the ranges reported above. The study also revealed that the protein contents of the Brightest Brilliant Rainbow and Titicaca varieties grown in Africa were significantly lower than those of the same varieties grown in Peru. In contrast, the protein content of the Kancolla variety grown in Africa was significantly higher than that of the variety grown in Peru [50].

The World Health Organization (WHO) has identified nine essential amino acids required for adult humans: phenylalanine, isoleucine, leucine, lysine, methionine, threonine, tryptophan, valine, and histidine [51]. According to the findings of [51], evaluation of four quinoa cultivars demonstrated that quinoa grain proteins contain considerable amounts of all essential amino acids. The mean values across cultivars in 2015 and 2016 showed leucine contents of 4.5 and 5.0 g, lysine contents of 3.5 and 4.5 g, and valine contents of 4.4 and 4.7 g, respectively. In addition, quinoa grain contains higher levels of essential amino acids and total amino acids than teff grain, except for leucine, which was found to be similar in both grains [9].

## 4. Materials and Methods

This section explains the research methodologies; where it was conducted; how the data was collected and analyzed; and the genotypes that were used in the research, with their origin presented briefly.

### 4.1. Descriptions of the Study Areas

The experiment was conducted during the 2015 main cropping season at six rain-fed locations. These locations represent various agro-ecologies of Ethiopia. The locations included are the Ethiopian Institute of Agricultural Research (EIAR) along with its five research centers at Kulumsa, Melkassa, Ziway, Holeta and Negelle Arsi and Haramaya University. The list of the locations used in experiment, with their climatic, soil type, rainfall and global position, are presented in Table 8.

### 4.2. The Experimental Materials

Five (5) quinoa genotypes obtained from FAO in Peru, 5 from Malawi, two previously introduced into Ethiopia, and one Amaranthus variety as a check were used; in total, 13 genotypes were used in this experiment (Table 9).

### 4.3. Experimental Design and Procedures

The experimental design used was a randomized complete block design (RCBD) with three replications. A plot size of 2 m width and 2 m length (4 m^2^) was used with flat beds for each genotype in each replication. Spacing of 50 cm and 10 cm between rows and plants, respectively, was employed. Planting was done in July 2015 after the onset of rainfall when there was enough soil moisture. A seeding rate per plot of 10 g or 25 kg/ha at a sowing depth of 1.5–2.0 cm were used, with other agronomic practices based on the recommendation given for each experimental site [52]. Thinning was performed one week after germination. A total of 50 kg/ha DAP and 100 kg/ha Urea were applied at planting and three to four weeks after emergence. Weeding was done as required and based on the level of infestations.

### 4.4. Data Collected

All the relevant agronomical, crop phenology, grain yield and biomass data were collected on a per plot basis. Five random plants were taken to measure plant height and estimate the yield component parameters. In addition, weather data, like the mean, maximum and minimum temperature, annual rainfall and other related information were collected during the growing seasons. The following data were collected from the experiment both on a per plot and per plant basis.

**Data recorded on a plot basis.**

1. **Date of emergence**: Number of days from sowing to emergence.
2. **Stand count at harvest**: The number of plants at harvest.
3. **Days to 50% flowering**: The number of days from emergence to the stage when 50% of the plants in a plot have produced flowers.
4. **Days to maturity**: The number of days from the date of emergence to the stage when 90% of plants have reached their physiological maturity.
5. **Grain filling period**: The period, in days, from flowering to maturity.
6. **Grain yield per plot**: Grain yield in grams, harvested from the two central rows, adjusted at 10% seed moisture content.
7. **Seed yield per hectare**: Seed yield obtained from each experimental plot and converted to get the seed yield per hectare.

The data for the following characteristics were recorded from ten randomly taken plants from each experimental plot and the average was considered on a per plant basis.

1. **Plant height**: The length from the root collar to the tip of the panicle of the plant. It was recorded at physiological maturity.
2. **Number of primary branches per plant**: The average number of primary branches of ten plants.
3. **Panicle length**: Measured from the main panicle base to tip at physiological maturity.
4. **Panicle width**: Records the maximum width of main panicle at physiological maturity.
5. **Number of panicles per plant**: The average number of panicles counted from the same sample plants.
6. **Grain yield per plant**: The average number of seeds obtained from each plant at harvest. At least 10 plants were included, and adjusted at 10% seed moisture content.

### 4.5. Data Analysis

#### 4.5.1. Analysis of Variance

Except for the scored data that were summarized using the standard descriptor for quinoa [53], all the collected data were analyzed using SAS statistical packages (SAS 9.4) for each location, and a combined analysis of variance was performed to see the differences among the genotypes in their overall performance across locations, following the standard procedure given by [54]. Mean separations were done for the statistically significant parameters based on DMRT of 1% and 5%. The data for each trait were subjected to a combined analysis of variance to estimate the effect of environment, genotype and genotype x environment interaction after using Bartlett’s homogeneity test (Table 10).

The model for simple analysis of variance:

Yijk = µ + Gi +Ej + GEij + Bjk + gijk

where µ is the mean; Gi = the effect of ith genotype; Ej = the effect of jth environment; GEij = the interaction of the ith genotype with the jth environment; Bjk = the effect of the kth replication in the j^th^ environment; and gijk = is the random error.

#### 4.5.2. AMMI Analysis

An AMMI analysis of the genotype x environment interaction was also employed [32]. The data for the seed yield were analyzed (Table 11) using this model because AMMI partitions the sum of squares into interaction principal component (IPC) axes.

The degree of freedom (DF) for the IPCA axes was calculated according to [32] with the following formula, DF = G + E − 1 − 2n, where G = the number of genotype 1, E = the number of environments, and n = the nth axis of IPCA.

#### 4.5.3. AMMI’s Stability Value

Following testing of significance of the GEI mean square over three replications for yield of varieties at location j, Yij were subjected to an AMMI stability analysis. The AMMI’s stability value (ASV) was calculated using the formula suggested by [45].

ASV_i_ = √ {[(SSIPCA1/SSIPCA2) × IPCA1_i_]^2^ + (IPCA2_i_)^2^}

where ASVi = AMMI’s stability value of genotype i; SSIPCA1 and SSIPCA2 = sum of squares explained by IPCA1 and IPCA2; and IPCA1i and IPCA2i = the score of genotype on the first and second interaction principal component axis, respectively.

#### 4.5.4. Protein and Amino Acid Analysis

The samples were taken from four genotypes, namely, Kancolla, Titicaca, Brightest Brilliant Rainbow and Amaranthus as a check. These genotypes were selected based on their performance across locations, as a pilot demonstration of quinoa’s nutritional potential. The total protein was determined using the method prescribed by the DIN Standards Committee [55]. The nitrogen content was determined with the combustion method using a DUMATHERM^®^ analyzer (C. Gerhardt GmGbH& Co. KG, Königs winter, Germany) according to the Dumas method [56].

A total of 100 mg of the crushed sample was weighed in triplicate. The universal protein-to-nitrogen conversion factor of 6.25 was used for the calculation of the protein content expressed in grams per 100 g of sample [56]. The amino acid analysis was carried out on a Perkin Elmer Series 200 HPLC system (Perkin Elmer, Waltham, MA, USA), connected to an API 2000 (AB Sciex, Framingham, MA, USA) triple quadruple mass spectrometer equipped with an Electro Spray Ionization (ESI) probe. A 10 µL aliquot was injected through an auto-sampler. Separation was achieved using a C18 column (Perkin Elmer, 220 mm × 4.6 mm × 5 mm). The mobile phase was composed of (A) water with 0.1% (*v*/*v*) formic acid and (B) methanol/water (50:50) with 0.1% (*v*/*v*) formic acid with a flow rate of 1 mL/min; the gradient was 0–1.0 min/2% (*v*/*v*) B, 1–10 min/2–80% (*v*/*v*) B, 10–12 min/80% (*v*/*v*) B, 12–13 min/80–2% (*v*/*v*) B and 12–18 min/2% (*v*/*v*) B. The ion source voltage was 5500 V; the temperature was 500 °C; the nebulizer gas was 207 kPa, and the heater gas was 379 kPa.

## 5. Conclusions

This study evaluated the effects of genotype, environment, and genotype × environment interaction (GEI) on quinoa yield and related traits and assessed the stability and adaptability of quinoa genotypes across diverse Ethiopian agro-ecologies. Significant differences were observed among the genotypes in grain yield, plant height, and days to maturity, confirming the strong influence of GEI on quinoa performance. The grain yield varied considerably across environments, with Kulumsa identified as the most favorable location for quinoa production. Among the tested genotypes, Brightest Brilliant Rainbow, Salcedo-INTA-Puno-Peru, and Multi-Hued consistently exhibited superior grain yield performance and broad adaptability, while Amarilla Sacaca, Brightest Brilliant Rainbow, and Salcedo-INTA-Puno-Peru showed relatively early maturity.

The AMMI and GGE biplot analyses with YSI, in general, identified Brightest Brilliant Rainbow and Salcedo-INTA-Puno-Peru as the most stable and widely adapted genotypes across environments, whereas Holeta and Kulumsa were recognized as favorable and representative testing environments. The stability analyses further indicated that Bio-bio, Multi-Hued, Cherry Vanilla, Amarilla Sacaca, Amarillla Maranganí, and Brightest Brilliant Rainbow possessed desirable stability characteristics across environments.

Considerable variation was also observed in the protein and essential amino acid contents among genotypes and environments, demonstrating the nutritional potential of quinoa under Ethiopian growing conditions. Brightest Brilliant Rainbow recorded the highest protein content, while several genotypes exhibited favorable essential amino acid profiles. Overall, the superior yield performance, stability, adaptability, early maturity, and nutritional quality of Brightest Brilliant Rainbow, Salcedo-INTA-Puno-Peru, and Multi-Hued highlight their strong potential for quinoa production and breeding programs aimed at improving food and nutritional security in Ethiopia. The results in this manuscript provide preliminary/baseline evidence suggesting that quinoa could be a potentially nutritionally rich crop in Ethiopia. However, future multi-year evaluations will be essential to confirm the stability and enable the release of superior, well-adapted varieties for production.

## Figures and Tables

**Table 1:** Mean performance of genotypes for yield and yield-related traits.

Genotype	PH	PBP	PL	PW	DM	NPP	SCH	GY
Blanca de Junín	90.92 cd	17.69 a	21.83 ab	13.11 a	99.99 cd	28.31 a	54.14 ab	1448.83 de
Amarilla Sacaca	87.22 d	16.30 ab	19.39 b	11.05 ab	92.88 d	25.62 ab	46.62 ab	1434.54 def
Amarilla Maranganí	103.04 ab	17.71 a	20.21 ab	14.66 a	105.13 ab	27.50 ab	45.45 ab	1429.69 def
Salcedo INIA	90.93 cd	17.77 a	20.59 ab	13.05 a	99.67 bc	26.17 ab	50.19 ab	1501.18 cd
Kancolla	104.47 a	15.69 ab	20.06 b	13.81 a	100.06 bc	23.35 ab	52.79 ab	1249.3 fg
Brightest Brilliant Rainbow	91.76 bcd	16.43 ab	19.49 b	10.73 ab	90.65 d	26.31 ab	44.34 b	1967.64 a
Bio-bio	89.53 cd	17.78 a	21.66 ab	11.18 ab	96.92 cd	27.79 ab	49.74 ab	1589.23 cd
Cherry Vanilla	100.25 abc	14.7 b	22.42 ab	7.72 b	96.5 cd	26.88 ab	48.00 ab	1294.2 efg
Multi-Hued	93.2 abcd	15.70 ab	22.20 ab	11.69 ab	95.09 cd	24.13 ab	52.94 ab	1671.24 bc
QQ74	92.64 bcd	17.68 a	21.50 ab	13.79 a	105.82 ab	22.55 b	45.71 ab	1585.73 cd
Titicaca	96.31 abcd	15.67 ab	23.31 ab	14.49 a	104.26 ab	27.30 ab	49.13 ab	1113.48 g
Salcedo-INTA-Puno-Peru	95.69 abcd	15.41 ab	20.06 b	11.58 ab	92.21 d	24.23 ab	48.75 ab	1777.53 b
Amaranths (Golden) (SC)	94.3 abcd	17.20 ab	24.91 a	12.08 a	106.84 a	25.34 ab	54.86 a	1225.96 g
Mean	93.93	16.68	21.24	12.33	98.37	25.95	49.48	1519.82
CV (%)	13.61	16.96	24.32	37.39	7.72	22.73	22.42	14.62

A common letter within a column indicates the values are not significantly different from each other (at *p* < 0.05) based on Duncan’s Multiple Range Test. CV = coefficient of variation; PH = plant height (cm); PBP = number of primary branches per plant; PL = panicle length (cm); PW = panicle width (cm); NPP = number of panicles per plant; SCH = stand count at harvest; DM = days to maturity; GY = grain yield (kg ha^−1^); SC = standard.

**Table 2:** Mean grain yield (kg ha^−1^) and test environment mean grain yield (kg ha^−1^) of 13 quinoa genotypes evaluated across six environments.

Genotype	Melkassa	Ziway	Negelle Arsi	Kulumsa	Holeta	Haramaya	GM	R
	E1	E2	E3	E4	E5	E6		
Blanca de Junín	1018	817	965	2035	2108	1750	1448	8
Amarilla Sacaca	961	779	1245	2000	1898	1946	1472	6
Amarillla Maranganí	859	765	1104	1877	1786	1695	1348	9
Salcedo INIA	889	713	1145	2136	2164	1960	1501	5
Kancolla	881	1274	818	1699	1429	1557	1276	10
Brightest Brilliant Rainbow	1021	1176	1332	2669	3033	2574	1968	1
Bio-bio	1018	1235	1128	2065	1972	2117	1589	4
Cherry Vanilla	753	751	925	1693	1639	1770	1255	11
Multi-Hued	1086	1300	1302	2132	2007	2201	1671	3
QQ74	786	733	1286	1986	1802	2101	1449	7
Titicaca	651	749	795	1292	1143	1648	1046	13
Salcedo-INTA-Puno-Peru	995	1224	1398	2161	2374	2321	1746	2
Amaranths (Golden) (SC)	925	727	784	1860	1447	1338	1180	12
EM	911	942	1094	1970	1908	1921		

EM = environment mean; GM = genotype mean; R = rank; SC = standard check. E1 = environment 1; E2 = environment 2; E3 = environment 3; E4 = environment 4; E5 = environment 5; E6 = environment 6.

**Table 3:** Analysis of variance of thirteen quinoa genotypes evaluated across six environments for eight traits.

Location	SoV	DF	PH	PBP	PL	PW	NPP	SCH	DM	GY
Melkassa	Genotype	12	353.9 ns	16.26 ns	34.43 ns	0.474 ns	23.08 ns	64.25 ns	35.08 *	46,998 ns
	Replication	2	306.4 ns	16.13 ns	32.05 ns	0.43 ns	13.55 ns	69.68 ns	17.76 ns	1757 ns
	Error	15	185.5	18.83	31.79	0.3073	16.43	47.68	9.587	23,036
	Mean		79.6	18.64	19.52	2.864	17.6	28.1	79.28	911
	CV		17.1	23.3	28.9	19.4	23	24.6	3.9	16.7
Ziway	Genotype	12	329.5 ns	8.47 ns	42.52 *	3.28 **	14.45 ns	223.79 *	6.077 ns	9135 **
	Replication	2	241.3 ns	18.30 ns	54.52 *	0.317 ns	40.46 ns	176.17 ns	22.03 *	1484 ns
	Error	15	252.6	8.127	11.57	0.6182	11.14	72.79	4.675	1205
	Mean		68.8	10.51	17.63	3.66	20.07	30.2	81.17	196.1
	CV		23.1	27.1	19.3	21.5	16.6	28.3	2.7	17.7
Negelle Arsi	Genotype	12	87 ns	9.93 ns	13.28 ns	0.593 ns	194.44 *	402.4 ns	33.5 ns	140,697 **
	Replication	2	1339.4 *	16.37 ns	124.14 *	0.78 ns	25.96 ns	115.4 ns	212.5 ns	3588 ns
	Error	15	299.3	18.32	28.79	0.601	84.73	200.8	103.1	39,672
	Mean		70.4	19.44	18.54	3.45	37.4	49.6	92	1094
	CV		24.6	22	28.9	22.5	24.6	28.5	11	18.2
Kulumsa	Genotype	12	1035.85 **	5.533 ns	1.21 ns	84.59 ns	32.28 ns	283.5 ns	393.27 **	305,812 **
	Replication	2	24.1 ns	12.58 ns	1.67 ns	156.14 *	12.45 ns	751.9 ns	140.59 ns	4923 ns
	Error	15	98.09	9.039	1.407	43.47	48.51	318.6	51.2	21,238
	Mean		135.9	15.87	4.7	24.9	28.3	95.9	113	1970
	CV		7.3	18.9	25	26.5	24.6	18.6	6.3	7.4
Holeta	Genotype	12	194.35 ns	19.22 ns	46.6 ns	4.17 ns	18.98 ns	76.5 ns	445.6 ns	681,655 **
	Replication	2	301.37 *	108.83 *	73.39 ns	2.99 ns	13.76 ns	218.6 ns	77.3 ns	93228 ns
	Error	15	85.32	18.55	44.56	2.176	18.94	130.1	258	81,299
	Mean		119.2	16.23	34.1	8.44	18.52	39.6	126.1	1908
	CV		7.7	26.5	19.6	17.5	23.5	28.8	12.7	14.9
Haramaya	Genotype	12	304.13 *	14.08 ns	162.51 ns	64.1 ns	129.84 *	49.18 ns	51.02 ns	346,942 ns
	Replication	2	53.32 ns	21.948 ns	251.49 ns	5164.73 **	34.28 ns	110.92 ns	184.88 *	622,208 *
	Error	15	92.71	7.412	77.42	81.03	39.17	66.24	43.88	151,543
	Mean		87.4	17.2	34.8	30.5	31.7	46.3	93.8	1921
	CV		11	15.8	25.3	29.5	19.8	17.6	7.1	20.3

** Highly significant at (*p* < 0.01); * significant at (*p* < 0.05); and ns—non-significant. DF = degrees of freedom; CV = coefficient of variance; PH = plant height (cm); PBP = number of primary branches per plant; PL = panicle length (cm); PW = panicle width (cm); NPP = number of panicles per plant; SCH = stand count at Harvest; DM = days to Maturity; GY = grain yield (kg ha^−1^).

**Table 4:** Combined analysis of variance of 13 quinoa genotypes evaluated across 6 environments for 8 traits.

		Mean Squares				
SoV	Environment (E)	Genotype (G)	G × E	Rep (E)	Error	CV
DF	5	12	60	2	76	
Traits						
**PH**	20,901.13 **	170.25 ns	291.60 **	95.27 ns	163.56	13.61
**PBP**	184.19 **	19.18 *	6.58 ns	23.33 ns	8.01	16.96
**PL**	3573.60 **	37.38 ns	22.79 ns	89.94 ns	26.67	24.32
**PW**	3788.40 **	23.17 ns	14.65 ns	476.99 **	21.28	37.39
**NPP**	1687.94 **	56.88 ns	37.68 ns	103.18 ns	34.78	22.72
**SCH**	17,370.83 **	215.90 ns	113.37 ns	424.43 ns	123.05	22.42
**DM**	8548.40 **	243.60 **	96.80 *	192.92 *	57.73	7.72
**GY**	7,122,173.46 **	860,406.24 **	94,419.08 **	43,220.30 ns	49,416.03	14.62

Note: ** Highly significant at *p* < 0.01; * significant at *p* < 0.05; and ns—non-significant. SoV = source of Variance; Rep (E) = replication within environment; DF = degree of freedom; CV = coefficient of variance; PH = plant height (cm); PBP = number of primary branches per plant; PL = panicle length (cm); PW = panicle width (cm); NPP = number of panicles per plant; SCH = stand count at harvest; DM = days to maturity; GY = grain yield (kg ha^−1^).

**Table 5:** ANOVA table for AMMI model for 13 quinoa genotypes grown in 6 environments.

SoV	DF	SS	MS	SS% Total	Explained % G × E	G × E Cumulative
Total	233	80,962,513	347,479			
Treatments	77	74,187,200	963,470 **	91.63		
Genotypes	12	13,328,021	1,110,668 **	16.46		
Environments	5	53,674,822	10,734,964 **	66.29		
Block	12	1,465,000	122,083	1.81		
Interactions	60	7,184,357	119,739 **	8.87		
IPCA1	16	4,638,157	289,885 **	5.73	64.56	82.54
IPCA2	14	1,292,166	92,298 ns	1.61	17.986	
Residuals	30	1,254,034	41,801 ns	1.55		
Error	95	5,310,313	55,898			

** Highly significant at (*p* < 0.01); SoV = source of variation; DF = degrees of freedom; SS = sum of squares; MS = mean sum of squares; SS% total = total percentage sum of squares; G × E = genotype-by-environment interaction. IPCA1 = interaction principal component analysis 1; IPCA2 = interaction principal component analysis 2.

**Table 6:** Four AMMI-selected genotypes per environment.

Environment	Mean	IPCA Score	1	2	3	4
Melkassa	911	14.17	G9	G6	G7	G12
Ziway	942	18.71	G9	G7	G12	G5
Negelle Arsi	1094	4.47	G6	G12	G9	G7
Holeta	1908	−24.13	G6	G12	G4	G1
Haramaya	1921	−5.48	G6	G12	G9	G10
Kulumsa	1970	−7.75	G6	G12	G4	G1
**Mean**	**1458**					

IPCA = interaction principal component analysis.

**Table 7:** AMMI stability value of thirteen genotypes tested across six environments.

Genotype	Yield Mean	Rank	IPCAg1	IPCAg2	ASV	Rank	YSI	Rank
Blanca de Junín	1449	8	−4.67857	10.78972	130.83	9	17	5
Amarilla Sacaca	1471	6	−1.46789	−1.52372	4.44	1	7	1
Amarillla Maranganí	1347	9	3.9851738	2.7226	26.63	4	13	3
Salcedo INIA	1501	5	−9.69974	2.67075	186.43	10	15	4
Kancolla	1276	10	16.22501	4.97923	500.00	13	23	6
Brightest Brilliant Rainbow	1968	1	2.70523	1.29684	32.79	6	7	1
Bio-bio	1589	4	3.05467	−1.99629	15.53	3	7	1
Cherry Vanilla	1255	11	2.38084	−2.83595	13.24	2	13	3
Multi-Hued	1671	3	3.97031	−4.07974	32.19	5	8	2
QQ74	1449	7	−2.70523	−10.15172	106.84	8	15	4
Titicaca	1046	13	13.30793	−10.04502	304.75	12	25	7
Salcedo-INTA-Puno-Peru	1746	1	−5.68824	−6.64882	74.13	7	8	2
Amaranths (Golden)	1180	12	8.05088	14.82213	267.47	11	23	6
Mean	1457.54							

**Table 8:** Temperature, rainfall, soil type, altitude, latitude and longitude of the locations.

Agro-Ecology	Haramaya University	Kulumsa ARC	Melkassa ARC	Ziway SRC	Negelle Arsi SRC	Holeta ARC
Altitude (m.a.s.l)	1980	2200	1550	1575	2043	2400
Latitude	9°26′ N	8°1′10′′ N	8°24′ N	8°20′ N	7°21′ N	9°00′ N
Longitude	42°3′ E	39°9′11′′ E	39°21′ E	39° E	38°42′ E	38° 30′ E
Annual rainfall (mm)	800	820	928	728	1020	1144
Minimum T (°C)	8.25	10.5	12.6	12.9	9.71	6
Maximum T (°C)	25.4	22.8	28.5	29.8	18	22
Soil type	Fluvisol	Luvisols	Nitosol	Nitosol	Nitosoil	Vertisoil

Note: ARC = agricultural research center and SRC = sub-research center.

**Table 9:** Description of quinoa genotypes used in the experiment.

Trt. No.	Genotypes	Code	Origin	Remark	Breeding History
1	Blanca de Junín	G1	Peru	FAO introduction	-
2	Amarilla Sacaca	G2	Peru	FAO introduction	-
3	Amarillla Maranganí	G3	Peru	FAO introduction	-
4	Salcedo INIA	G4	Peru	FAO introduction	-
5	Kancolla	G5	Peru	FAO introduction	-
6	Brightest Brilliant Rainbow	G6	Oregon, US	Imported from Malawi	Bred by Frank Morton of Wild Garden Seeds
7	Bio-bio	G7	Chile	Imported from Malawi	-
8	Cherry Vanilla	G8	Oregon, US	Imported from Malawi	Bred by Frank Morton of Wild Garden Seeds
9	Multi-Hued	G9	British Columbia, Canada	Imported from Malawi	-
10	QQ74	G10	Denmark	Imported from Malawi	Heat-tolerant Chilean landrace
11	Titicaca	G11	Peru	Previous introduction	Bred by Sven-Erik Jacobsen
12	Salcedo-INTA-Puno-Peru	G12	Denmark	Previous introduction	-
13	Amaranthus	G13	Uganda	Check	Cultivar

**Table 10:** Analysis of variance and mean square value for genotype, environment and G × EI.

Source of Variance	DF	Mean Square
Replication (r)	r − 1	Msr
Environment (e)	e − 1	Mse
Genotype (g)	g − 1	Msg
Genotype × Environment	(e − 1) (g − 1)	Msexg
Error (e)	(r − 1) (g − 1)	Ms δ^2^ e

**Table 11:** ANOVA table for AMMI model.

SoV	DF	SS	MS	SS% Total	Explained % G × E	G × E Cumulative
Total	233	80,962,513	347,479			
Treatments	77	74,187,200	963,470 **	91.63		
Genotypes	12	13,328,021	1,110,668 **	16.46		
Environments	5	53,674,822	10,734,964 **	66.29		
Block	12	1,465,000	122,083	1.81		
Interactions	60	7,184,357	119,739 **	8.87		
IPCA1	16	4,638,157	289,885 **	5.73	64.56	82.54
IPCA2	14	1,292,166	92,298 ns	1.61	17.986	
Residuals	30	1,254,034	41,801 ns	1.55		
Error	95	5,310,313	55,898			

** Highly significant at (*p* < 0.01); SoV = source of variation; DF = degrees of freedom; SS = sum of squares; MS = mean sum of squares; SS% total = total percentage sum of squares; G × E = genotype-by-environment interaction. IPCA1 = interaction principal component analysis 1; IPCA2 = interaction principal component analysis 2.

## Data Availability

The original contributions presented in this study are included in the article. Further inquiries can be directed to the corresponding authors.

## Abbreviations

The following abbreviations are used in this manuscript:

AEZs	Agro-Ecological Zones
AEC	Average Environment Coordination
AMMI	Additive Main Effects and Multiplicative Interaction
ANOVA	Analysis of Variance
CV	Coefficient of Variance
EIAR	Ethiopian Institute of Agricultural Research
EAA	Essential Amino Acids
ESI	Electrospray Ionization
FAO	Food and Agriculture Organization
GEI	Genotype-by-Environment Interaction
IPC	Interaction Principal Component
ISSR	Inter Simple Sequence Repeat
Masl	Meter Above Sea Level
MET	Multi-Environment Trial
MoA	Ministry of Agriculture
PCA	Principal Component Analysis
RASP	Random Amplified Sequence Polymorphism
RCBD	Randomized Complete Block Design
TDF	Total Dietary Fiber
TPE	Target Population of Environment
WHO	World Health Organization

## Appendix A

### Appendix A.1. Scored Data over Locations Summarized Using Standard Descriptor for Quinoa

**Genotype**	**Growth Habit**	**Panicle Density**	**Panicle Shape**
Blanca de Junín	Branched to bottom third	Compact	Intermediate
Amarilla Sacaca	Branched to second third	Intermediate	Intermediate
Amarillla Maranganí	Branched to second third	Intermediate	Intermediate
Salcedo INIA	Branched to second third	Compact	Intermediate
Kancolla	Branched to second third	Intermediate	Intermediate
Brightest Brilliant Rainbow (BBR)	Branched to second third	Compact	Intermediate
Bio-bio	Branched to second third	Intermediate	Intermediate
Cherry Vanilla	Branched to second third	Intermediate	Amarantiform
Multi-Hued	Branched to bottom third	Compact	Glomerulate
QQ74	Branched to second third	Intermediate	Intermediate
Titicaca	Branched to second third	Intermediate	Intermediate
Salcedo-INTA-Puno-Peru	Branched to second third	Intermediate	Intermediate
Amaranths (Golden)	Branched to bottom third	Compact	Amarantiform

**Panicle shape:**

1. Glomerulate = Glomerules are inserted in the primary axis showing a globose shape.
2. Intermediate = Showing both shapes.
3. Amarantiform = Glomerules are inserted directly in the secondary axis and have an elongated shape.

**Growth habit:**

1. Simple.
2. Branched to bottom third.
3. Branched to second third.
4. Branched with main panicle undefined.

**Panicle density:**

1. Lax.
2. Intermediate.
3. Compact.

### Appendix A.2. Protein and Amino Acid Profiling of Three Quinoa and One Amaranth Varieties Grown in Five Environments

**Amino Acid Profile**													
	**Genotype**	**Protein**	**Aspartic Acid**	**Threonine**	**Serine**	**Glutamic Acid**	**Proline**	**Glycine**	**Alanine**	**Valine**	**Methionine**	**Isoleucine**	**Leucine**
**Melkassa**	**BBR**	17.8	1.55	0.62	0.81	2.63	0.65	1.06	0.76	0.80	0.24	0.72	1.14
	**Titicaca**	16.1	1.53	0.59	0.92	2.49	0.63	1.11	0.73	0.91	0.25	0.70	1.08
	**Amaranth**	17.0	1.51	0.58	0.98	2.69	0.68	1.35	0.64	0.85	0.25	0.72	1.03
**Holeta**	**Amaranth**	16.1	1.46	0.56	0.89	2.69	0.67	1.30	0.61	0.84	0.26	0.69	0.98
	**Titicaca**	17.3	1.55	0.62	0.96	2.44	0.64	1.15	0.77	0.79	0.21	0.71	1.07
	**Kancolla**	16.2	1.60	0.86	1.36	2.49	0.83	1.29	0.97	1.00	0.21	1.04	1.27
	**BBR**	15.2	1.49	0.79	1.16	2.29	0.76	1.04	0.81	0.88	0.18	0.69	1.14
**Ziway**	**BBR**	16.8	1.70	0.88	1.56	2.59	0.85	1.33	0.92	1.05	0.19	0.78	1.27
**Kulumsa**	**BBR**	14.8	1.45	0.78	1.04	2.35	0.74	1.09	0.78	0.90	0.14	0.68	1.14
	**Kancolla**	15.9	1.68	0.85	1.27	2.49	0.85	1.21	0.88	1.01	0.20	0.78	1.26
	**Amaranth**	16.2	1.61	0.83	1.35	2.96	0.88	1.44	0.79	0.90	0.20	0.76	1.18
	**Titicaca**	16.9	1.67	0.86	1.22	2.87	0.86	1.21	0.89	0.97	0.21	0.76	1.29
**Negelle Arsi**	**Amaranth**	17.0	1.68	0.83	1.74	2.94	1.23	1.57	0.92	0.96	0.29	0.74	1.18
	**BBR**	15.8	1.55	0.74	1.29	2.37	0.99	1.26	0.91	0.88	0.24	0.68	1.22
	**Titicaca**	16.3	1.59	0.77	1.31	2.54	1.16	1.21	0.91	0.93	0.23	0.72	1.22

**Amino Acid Profile** **Continued**												
		**Tyrosine**	**Phenylamine**	**Ornithine**	**γ-Aminobultyric Acid**	**Lysine**	**Histdine**	**Arginine**	**Taurine**	**Hydroxyproline**	**Cystine**	**Hydroxylsine**
**Melkassa**	**BBR**	0.45	0.64	<0.05	<0.05	0.87	0.43	1.51	<0.05			<0.05
	**Titicaca**	0.51	0.65	<0.05	<0.05	0.79	0.48	1.29	<0.05	<0.05	0.15	<0.05
	**Amaranth**	0.53	0.72	<0.05	<0.05	0.90	0.35	1.31	<0.05	<0.05	0.21	<0.05
**Holeta**	**Amaranth**	0.52	0.69	<0.05	<0.05	0.94	0.35	1.34	<0.05	<0.05	0.20	<0.05
	**Titicaca**	0.48	0.65	<0.05	<0.05	0.86	0.43	1.30	<0.05	<0.05	0.15	<0.05
	**Kancolla**	0.44	0.59	<0.05	<0.05	1.10	0.57	1.39	<0.05	<0.05	<0.05	<0.05
	**BBR**	0.42	0.54	<0.05	<0.05	1.06	0.46	1.31	<0.05	<0.05	<0.05	<0.05
**Ziway**	**BBR**	0.57	0.68	<0.05	<0.05	1.08	0.68	1.45	<0.05	<0.05	<0.05	<0.05
**Kulumsa**	**BBR**	0.39	0.58	<0.05	<0.05	1.07	0.51	1.32	<0.05	<0.05	<0.05	<0.05
	**Kancolla**	0.48	0.64	<0.05	<0.05	0.99	0.54	1.46	<0.05	<0.05	<0.05	<0.05
	**Amaranth**	0.51	0.63	<0.05	<0.05	1.08	0.48	1.52	<0.05	<0.05	<0.05	<0.05
	**Titicaca**	0.47	0.66	<0.05	<0.05	1.11	0.52	1.64	<0.05	<0.05	<0.05	<0.05
**Negelle Arsi**	**Amaranth**	0.58	0.73	<0.05	<0.05	1.34	0.86	1.74	<0.05		<0.05	<0.05
	**BBR**	0.37	0.57	<0.05	<0.05	1.25	0.76	1.49	<0.05	<0.05	<0.05	
	**Titicaca**	0.45	0.63	<0.05	<0.05	1.25	0.77	1.60	<0.05	<0.05	<0.05	<0.05
